# Immunoglobulin κ drives liver regeneration by stabilizing MYLK to maintain cytoskeletal integrity and promote hepatocyte proliferation

**DOI:** 10.1515/jtim-2026-0054

**Published:** 2026-06-13

**Authors:** Huining Gu, Sha Yin, Feng Liu, Jingjing Guo, Jiongming Yang, Wanxuan Zhang, Teng Li, Shenghua Zhang, Huiying Rao, Jianze Wang, Zhaofei Liu, Weiyan Xu, Shaoping She, Xiaoyan Qiu, Jing Huang

**Affiliations:** Department of Immunology, School of Basic Medical Sciences, and NHC Key Laboratory of Medical Immunology, Peking University, Beijing, China; Peking University People's Hospital, Peking University Hepatology Institute, Beijing, China; Department of Radiation Medicine, School of Basic Medical Sciences, Peking University and Department of Nuclear Medicine, Peking University Cancer Hospital and Institute, Beijing, China; PUHSC Primary Immunodeficiency Research Center, School of Basic Medical Sciences, Peking University, Beijing, China

**Keywords:** hepatocyte, immunoglobulin κ, proliferation, liver regeneration, cytoskeleton, myosin light chain kinase

## Abstract

**Background and Objectives:**

Liver regeneration necessitates coordinated cytoskeletal dynamics, yet the key molecular orchestrators remain incompletely defined. Intriguingly, recent studies reveal a novel role for hepatocyte-derived Igκ in promoting hepatocyte survival during liver injury. However, the precise mechanisms by which immunoglobulin κ (Igκ) governs liver regeneration and its potential as a central coordinator remain elusive. This study aims to elucidate the role of Igκ in liver regeneration following injury and to define the underlying molecular mechanisms.

**Methods:**

Igκ expression was assessed in liver biopsies from patients with drug-induced liver injury (DILI) using immunohistochemistry. To investigate its role in regeneration, hepatocyte-specific Igκ-knockout mice were subjected to partial hepatectomy (PHx) or acetaminophen (APAP)-induced acute liver injury. Functional and mechanistic studies were performed in the normal human hepatocyte cell line THLE-2 through Igκ knockdown or overexpression, combined with multi-omics profiling, immunoprecipitation, and mass spectrometry. The therapeutic potential of Igκ was validated by AAV8-mediated hepatic delivery of Igκ in knockout mice.

**Results:**

Igκ expression is significantly upregulated in both human DILI patients and murine injury models upon hepatic damage. Hepatocyte-specific Igκ deletion impaired liver regeneration, characterized by disrupted cytoskeletal organization and diminished hepatocyte proliferation. Mechanistically, Igκ directly binds to myosin light chain kinase (MYLK), shielding it from K48-linked ubiquitination and proteasomal degradation, thereby preserving cytoskeletal integrity and facilitating Yes-associated protein (YAP) nuclear translocation to activate proliferative pathways. Crucially, AAV8-mediated hepatic Igκ delivery in knockout mice rescues MYLK protein levels, restores cytoskeletal integrity, and promotes liver regeneration.

**Conclusions:**

Our study identifies Igκ as a pivotal regulator of liver regeneration by stabilizing MYLK to maintain cytoskeletal dynamics and potentiate YAP-dependent proliferative signaling, thereby proposing a potential therapeutic strategy for enhancing hepatic repair.

## Introduction

The liver demonstrates robust regenerative capacity through hepatocyte proliferation, enabling critical interventions such as partial hepatectomy and split-liver transplantation for treating advanced liver disease and certain malignancies.^[1,2]^ Nevertheless, hepatic disorders remain a major and growing global health burden,^[3]^ underscoring the urgent need to develop regenerative enhancement therapies for patients with compromised regenerative capacity.

Liver regeneration primarily occurs through the proliferation of quiescent mature hepatocytes that re-enter the cell cycle post-injury/resection, regulated by biochemical signals and mechanical cues.^[1,4]^ The actin cytoskeleton is a critical regulator in this process, generating mechanical forces *via* the actomyosin system and modulates cellular behavior through signaling cascades, such as Yes-associated protein (YAP) nuclear translocation.^[5,6]^ During regeneration, hepatocytes dynamically sense and remodel their mechanical microenvironment, encompassing increased portal pressure, nascent cell-cell contacts, and extracellular matrix remodeling. These adaptive changes initially promote regenerative responses while later restraining excessive hepatocyte proliferation upon functional recovery.^[5,7]^ However, the key molecular mediators translating these mechanical and biochemical cues into the controlled regenerative program remain incompletely characterized.

Immunoglobulins (Igs) are tetrameric molecules composed of heavy and light chains, with light chains classified into κ and λ subtypes.^[8]^ Typically, heavy and light chains are synthesized asynchronously on separate ribosomes, with light chains exceeding heavy chain production by 10%–40% in B cells.^[9,10]^ Unbound excess light chains can be released into the systemic circulation in a free form, referred to as free light chains (FLCs), traditionally considered biologically inert byproducts of antibody assembly. However, emerging evidence indicates that FLCs participate in diverse immunological processes, functioning as signaling effectors or immunomodulatory molecules.^[11]^ Our previous work demonstrated widespread expression of Igκ-FLC in cardiac, hepatic, pulmonary, and renal tissues, where it exerts critical biological functions.^[12]^ Specifically, the Vκ4–1/Jκ3-Igκ functions as a novel extracellular matrix protein that binds integrin β1 to activate focal adhesion kinase (FAK) signaling, thereby promoting cell proliferation and migration.^[13]^ Furthermore, we recently identified hepatocyte-derived Igκ as a regulator of hepatocyte survival that mitigates concanavalin A (ConA)-induced liver injury.^[14]^ However, the potential role of Igκ in modulating liver regeneration remains unexplored.

In this study, we demonstrate that Igκ expression is upregulated during the recovery phase following liver injury. Deletion of Igκ in hepatocytes significantly impairs their proliferative and migratory capacity, thereby inhibiting liver regeneration after injury. Mechanistically, we show that hepatocyte-derived Igκ stabilizes myosin light chain kinase (MYLK), thereby promoting cytoskeletal integrity and YAP-mediated proliferation in response to physical or chemical liver damage, ultimately accelerating liver repair and regeneration. Collectively, our findings uncover a novel mechanism by which hepatocyte-derived Igκ contributes to liver regeneration. These results redefine Igκ-FLC as a multifunctional regulator essential to diverse cellular activities and fundamental biological processes.

## Materials and methods

### DILI patients and specimens

Liver biopsy specimens were obtained from six patients diagnosed with DILI and three healthy liver tissue samples at Peking University People's Hospital. The etiologies included acute liver injury due to acetaminophen (APAP) overdose and liver damage associated with long-term use of traditional Chinese medicine. The studies involving human tissues were approved by the Human Research Ethics Committee of Peking University People's Hospital (No. 2022PHB088).

### Animal studies

To generate conditional knockout mice, *Igκ^fl/fl^* mice were crossed with Albumin-cre transgenic mice, resulting in *Alb-cre^−^: Igκ^fl/fl^* (WT) mice and *Alb-cre^+^: Igκ^fl/fl^* (cKO) mice. All experimental procedures were approved by the Peking University Laboratory Animal Research Committee and conducted by the Institutional Animal Care and Use Committee guidelines of China (Approval No. DLASBD0693).

The PHx procedure was performed as follows: mice were anesthetized with tribromoethanol, and the median and left lateral liver lobes were ligated and resected. All surgeries were conducted under sterile laminar flow conditions, and post-operative mice were housed in sterile micro-isolator cages. APAP was dissolved in warm saline and administered *via* intraperitoneal injection at a dose of 300 mg/ kg. For viral intervention groups, AAV8 vectors expressing Igκ (Hanbio Biotechnology Co., Ltd., Shanghai, China) were injected *via* the tail vein 14 days prior to PHx. Mice were euthanized at predetermined time points or upon reaching humane endpoints.

### Statistical analysis

Statistical analyses were performed using GraphPad Prism software (La Jolla, CA). The correlation between two variables was analyzed by the Spearman correlation coefficient. Differences between the two groups were assessed with the two-tailed t-tests. For comparisons among three or more groups, one-way ANOVA followed by two-tailed t-tests was used. Statistical significance was defined as follows: ^****^P < 0.0001, ^***^P < 0.001, ^**^P < 0.01, and ^*^P < 0.05.

For further details regarding the materials and methods used, please refer to the supplementary information.

## Results

### Igκ is physiologically expressed in hepatocytes and upregulated during liver regeneration

**Figure 1:**
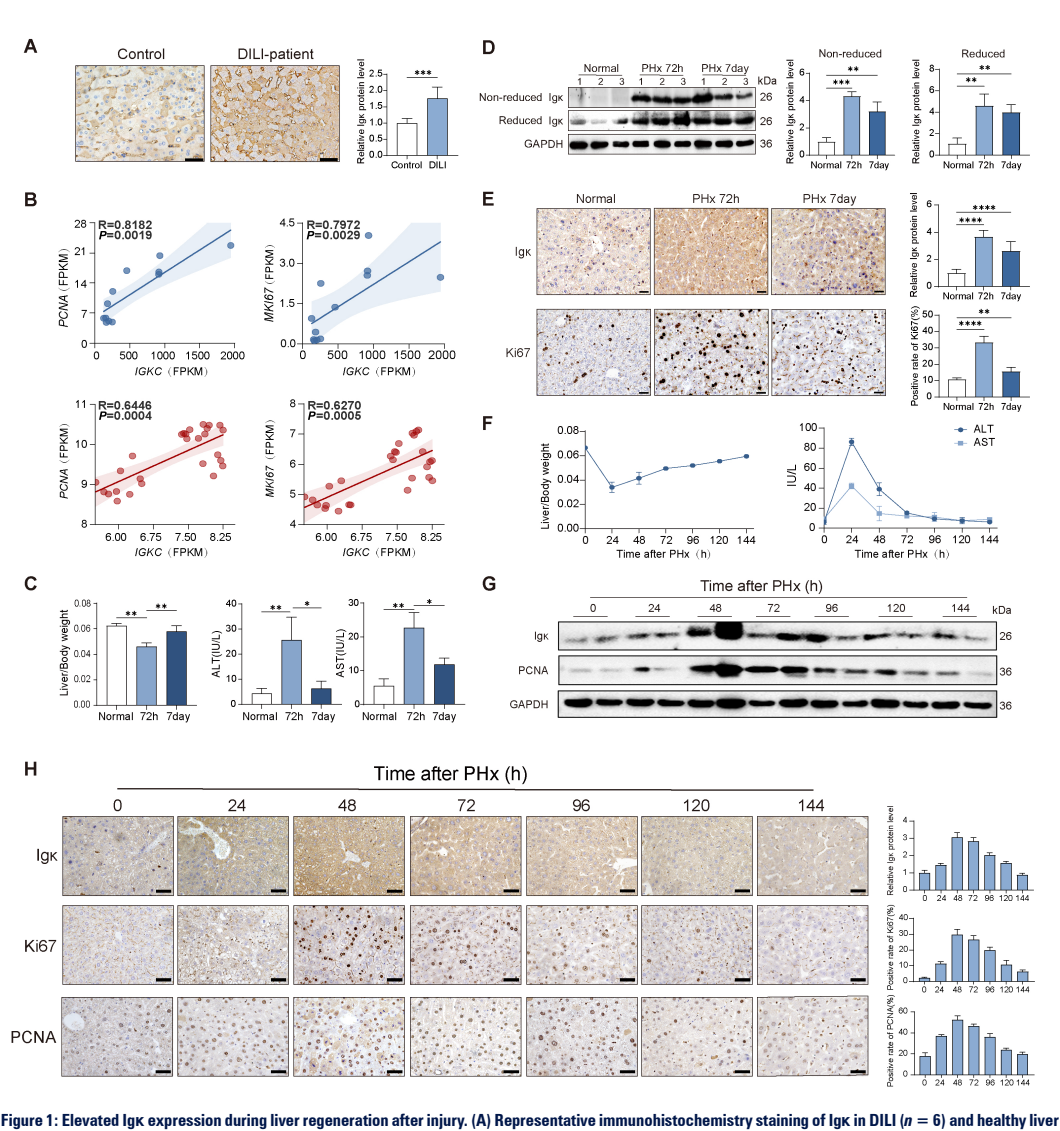
Elevated Igκ expression during liver regeneration after injury. (A) Representative immunohistochemistry staining of Igκ in DILI (*n* = 6) and healthy liver tissue (*n* = 3) is shown. The statistical analysis comparing the two groups is presented on the right. Scale bars, 50 μm. (B) Transcript-level correlation of IGKC with proliferation markers PCNA and MKI67 in hepatic injury models from GEO datasets. Liver biopsies from liver transplant recipients (upper, GSE186580). Liver tissues from patients with acute liver failure and normal liver donors (lower, GSE38941). (C) Liver-to-body weight ratio, serum ALT and AST levels in 70% PHx model (WT mice) were measured. (D) Western blot analysis of Igκ expression in liver tissues of WT mice at different time points post-PHx. Statistical results are shown on the right. (E) Representative immunohistochemistry staining of Igκ and Ki67 in liver tissues of WT mice. Scale bars, 30 μm. Statistical analysis is presented on the right. (F) Liver-to-body weight ratio, serum ALT and AST levels in 70% PHx model (μMT mice) were measured. (G) Western blot analysis of Igκ and PCNA expression in liver tissues of μMT mice at different time points post-PHx. (H) Representative immunohistochemistry staining of Igκ, Ki67 and PCNA in liver tissues of μMT mice after PHx at indicated time points. Scale bars, 50 μm. Data are presented as the mean ± SD. Statistical results are shown on the right. ^*^P < 0.05, ^**^P < 0.01, ^***^P < 0.001, ^****^P < 0.0001. WT: wild-type; DILI: drug-induced liver injury; PCNA: proliferating cell nuclear antigen; ALT: alanine aminotransferase; AST: aspartate aminotransferase.

Although B cell-derived immunoglobulins are well established in adaptive immunity, emerging evidence suggests that non-immune cells, including hepatocytes, can also express immunoglobulin components. Previous studies have established Igκ expression in hepatocytes of wild-type (WT) and B-cell-deficient μMT mice, implicating a non-canonical role of Igκ in liver biology.^[14]^ To determine whether this phenomenon extends to humans, we analyzed public single-cell RNA sequencing data and identified Igκ transcripts in human hepatocytes (Supplementary Figure S1A). We further validated this finding in the normal human hepatocyte cell line THLE-2, confirming Igκ expression at both mRNA and protein levels by RT-PCR and western blotting (Supplementary Figure S1B & C). Immunofluorescence staining revealed predominant cytoplasmic localization of Igκ (Supplementary Figure S1D). Given our previous work linking Igκ expression in murine hepatocytes to cell survival and ConA-induced liver injury,^[14]^ we sought to investigate its relevance in human liver disease. Immunohistochemical analysis of liver biopsies from patients with drug-induced liver injury showed significant upregulation of Igκ compared to healthy liver tissues (Figure 1A). Furthermore, systematic bioinformatics analysis of GEO datasets revealed a significant positive correlation between *IGKC* expression and proliferation-associated genes *PCNA* and *MKI67* in both liver transplant recipients and patients with acute liver failure (ALF)(Figure 1B), suggesting a potential relationship between Igκ and the process of liver regeneration following injury.

To further delineate Igκ dynamics during liver regeneration, we utilized a 70% PHx model in C57/BL6J mice, a well-established system for studying liver regeneration after acute injury. As expected, the liver-to-body weight ratio and serum levels of alanine aminotransferase (ALT) and aspartate aminotransferase (AST), indicative of of hepatocellular damage, peaked at 72 h post-PHx and returned to baseline by 7 days (Figure 1C). During this regenerative phase, Igκ protein expression were significantly upregulated, predominantly in its free form, and exhibited a temporal pattern that closely aligned with hepatic recovery (Figure 1D). Immunohistochemistry (IHC) analysis further revealed that Igκ expression paralleled the proliferation marker Ki67 as an indicator for the onset of the cellular regenerative response (Figure 1E), suggesting a possible involvement of Igκ in the proliferative response of hepatocytes.

Notably, although Igκ is traditionally considered a product of B lymphocytes and plasma cells, we questioned whether its expression during liver regeneration depends on adaptive immunity. To address this, we subjected B cell-deficient μMT mice to PHx. As shown in Figure 1F, these μMT mice displayed comparable liver injury and regeneration phenotypes to wild-type controls following PHx, including an initial decline in liver-to-body weight ratio with subsequent recovery, accompanied by transient elevations in serum ALT and AST levels. Importantly, Igκ expression in μMT mice remained temporally synchronized with the proliferation markers proliferating cell nuclear antigen (PCNA) and Ki67 throughout the regeneration process (Figure 1G & H). Together, these findings suggest that hepatocyte-derived Igκ may participate in a regeneration-associated program that operates independently of adaptive immunity, pointing to a non-canonical function of Igκ in liver regeneration.

### Hepatocyte-specific depletion of Igκ impairs liver regeneration

**Figure 2:**
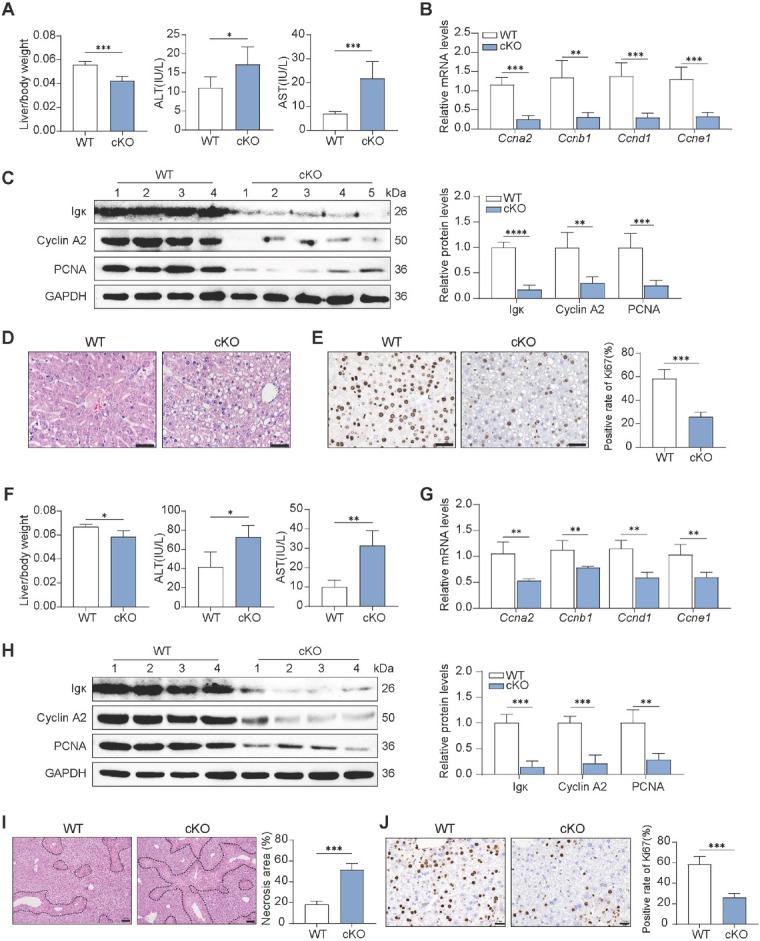
Hepatocyte-specific depletion of Igκ impairs liver regeneration in PHx (A-E) and APAP-induced acute liver injury (F-J) models. (A) & (F) Liver-to-body weight ratio, serum ALT and AST levels were measured. (B) & (G) The mRNA levels of cell cycle genes (Ccna2, Ccnb1, Ccnd1 and Ccne1) in WT and cKO liver tissues were analyzed by RT-qPCR. (C) & (H) Protein expression of Igκ, Cyclin A2, and PCNA in WT and cKO livers were assessed by western blotting. Statistical analysis is presented on the right. (D) & (I) Representative images of H & E staining (scale bars: 50 μm for D, 100 μm for I) in WT and cKO liver tissues. Quantification of necrosis area was shown in right panels. (E) & (J) Representative images of IHC staining for Ki67 (scale bars: 50 μm for E, 30 μm for J) in WT and cKO liver tissues. Quantification of Ki67-positive cells was shown in right panels. Data are presented as the mean ± SD. ^*^P < 0.05, ^**^P < 0.01, ^***^P < 0.001, ^****^P < 0.0001. WT: wild-type; PCNA: proliferating cell nuclear antigen; ALT: alanine aminotransferase; AST: aspartate aminotransferase; IHC: immunohistochemistry; cKO: conditional knockout.

To establish the functional significance of hepatocyte-derived Igκ in liver regeneration, hepatocyte-specific Igκ knockout mice (*Alb-cre^+^: Igκ^fl/fl^*, cKO) were generated by crossing *Igκ^fl/fl^* mice with albumin-cre transgenic mice. Having previously verified hepatocyte-specific Igκ deletion,^[14]^ we subjected cKO and wild-type controls (*Alb-cre^-^: Igκ^fl/fl^*, WT) to 70% PHx. The cKO mice exhibited markedly impaired regenerative capacity compared to control littermates 72 h post-PHx, evidenced by reduced liver-to-body weight ratios and persistently elevated serum ALT and AST levels (Figure 2A). Molecular profiling revealed that the levels of cell cycle-related genes (*Ccna2*, *Ccnb1*, *Ccnd1*, and *Ccne1*) were significantly downregulated in cKO livers (Figure 2B), accompanied by diminished protein levels of Cyclin A2 and PCNA (Figure 2C). Consistent with these findings, the livers of cKO mice showed disrupted lobular architecture on H & E staining and fewer Ki67-positive cells compared with WT mice (Figure 2D & E). To further validate these findings in clinically relevant models, mice received intraperitoneal administration of a sublethal APAP dose (300 mg/ kg) to induce hepatotoxicity, followed by euthanasia at 48 h post-exposure. Similar to the findings in the PHx model, cKO mice exhibited exacerbated liver injury accompanied by a significantly attenuated liver regeneration phenotype (Figure 2F-H). Additionally, increased hepatic necrosis areas and fewer Ki67-positive cells in the livers of cKO mice were observed compared with WT mice (Figure 2I & J). Taken together, these findings demonstrate that hepatocytic Igκ deficiency impairs liver injury repair and regeneration after acute injury induced by mechanical or chemical factors.

### Igκ regulates hepatocyte proliferation and migration in vitro

**Figure 3:**
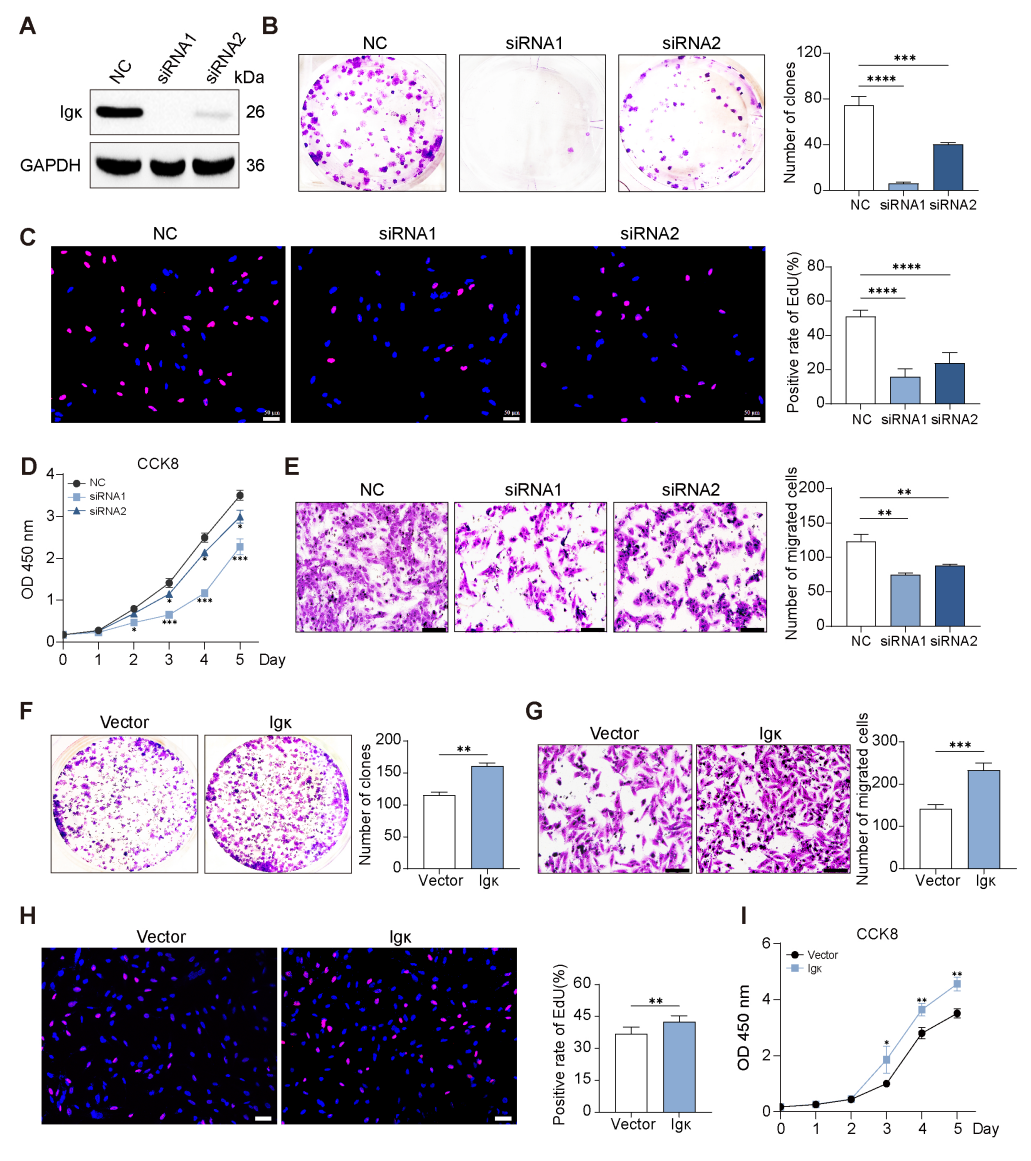
Igκ promotes proliferation and migration of THLE-2 cells. (A) Western blot analysis of Igκ protein levels in THLE-2 cells validates knockdown efficiency. (B) & (F) Colony formation assay was conducted to detect the proliferation capacity of THLE-2 cells after Igκ knockdown (B) or overexpression (F). The number of colonies was quantified (right) (*n* = 3). (C) & (H) EdU incorporation assay were performed to assess the proliferative capacity of THLE-2 cells after Igκ knockdown (C) or overexpression (H). EdU, red. The positive rate of EdU incorporation is shown (right) (*n* = 6). Scale bar: 50 μm. (D) & (I) CCK8 assay was performed to measure the proliferative capacity of THLE-2 cells after Igκ knockdown (D) or overexpression (I). (E) & (G) Transwell migration assays were used to evaluate the migration ability of THLE-2 cells with Igκ knockdown (E) or overexpression (G). The number of migrated cells was quantified (right) (*n* = 6). Scale bar: 50 μm. Data presented as the mean ± SD. ^*^P < 0.05, ^**^P < 0.01, ^***^P < 0.001, ^****^P < 0.0001.

To investigate the functional role of Igκ in hepatocyte proliferation, two siRNAs targeting the constant region of Igκ were designed to knock down Igκ expression. Successful knockdown efficiency was confirmed *via* western blotting (Figure 3A). siRNA-mediated Igκ silencing significantly inhibited THLE-2 cell proliferation, as demonstrated by diminished colongenic capacity by colony formation assay (Figure 3B). Furthermore, EdU incorporation assay revealed impaired DNA synthesis, along with reduced CCK-8 absorbance indicating decreased metabolic activity in Igκ-depleted cells (Figure 3C & D), confirming the inhibitory effect of Igκ knockdown on cell proliferation *in vitro*. Additionally, transwell assays demonstrated a marked reduction in migratory ability of THLE-2 cells upon Igκ knockdown compared to scrambled siRNA controls (Figure 3E). Conversely, overexpression of Igκ remarkably promoted THLE-2 cell proliferation and migration (Figure 3F-I). These results collectively demonstrate that Igκ is critical for regulating hepatocyte proliferation and migration.

### Igκ deficiency leads to hepatic cytoskeletal dysregulation during liver regeneration

**Figure 4:**
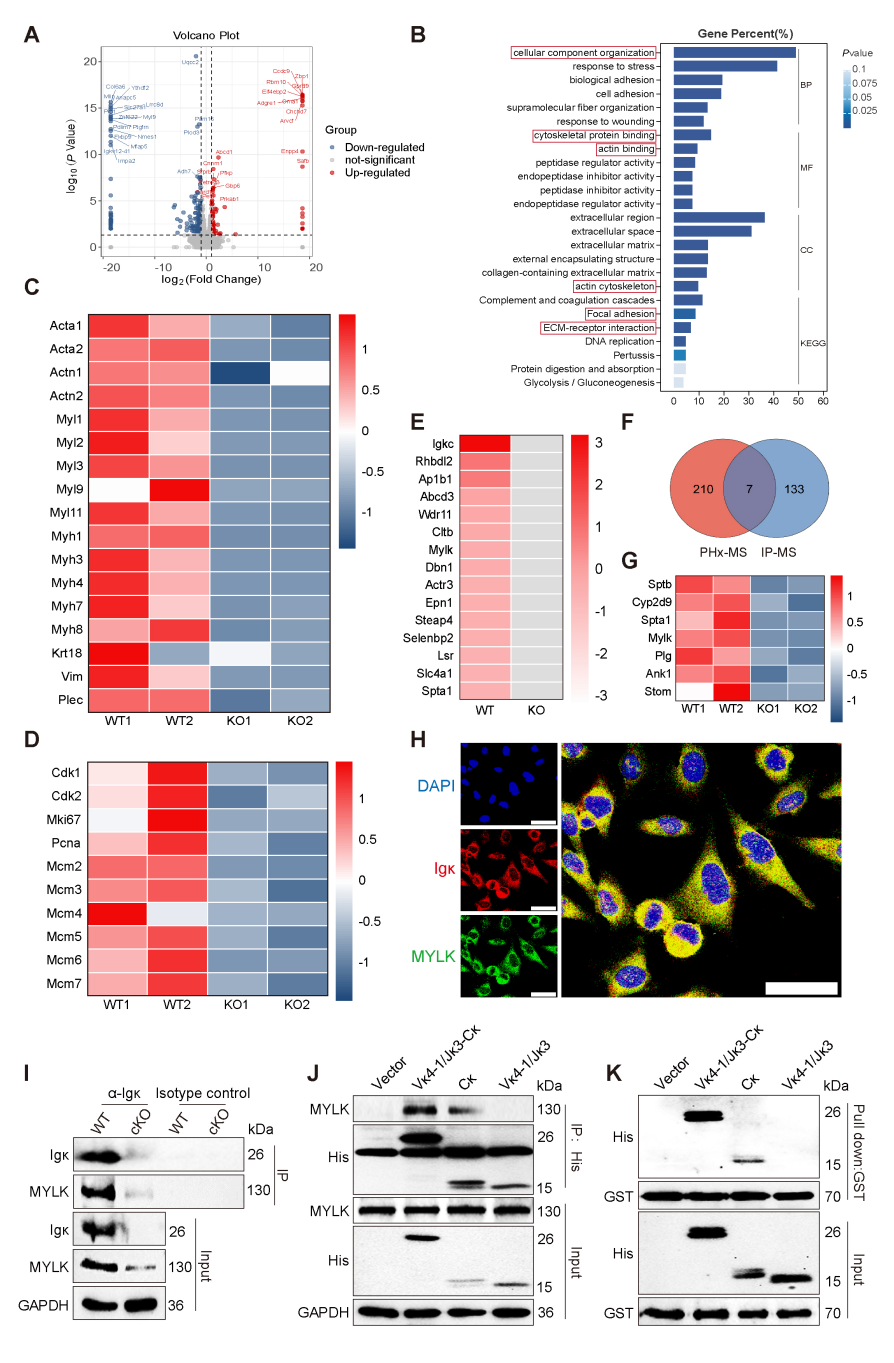
Igκ deficiency leads to hepatic cytoskeletal disorders during acute injury. (A) Volcano plot of dysregulated hepatic proteins in WT *vs*. cKO mice from label-free LC-MS/MS proteomic (red, upregulated; blue, downregulated). (B) Functional annotation of differentially expressed proteins *via* GO/KEGG enrichment analysis. (C) Heatmap of differentially expressed cytoskeleton-associated proteins. (D) Heatmap of differentially expressed proliferation-related proteins. (E) Heatmap of top 15 candidate proteins identified by IP-MS. (F) Venn diagram showing 7 overlapping proteins between LC-MS/MS and IP-MS datasets. (G) Heatmap of the 7 overlapping proteins identified in (F). (H) Confocal immunofluorescence demonstrating colocalization of Igκ (red) and MYLK (green) in THLE-2 cells. Scale bars: 50 μm. (I) Co-IP of Igκ and MYLK interaction in WT and cKO liver lysates using anti-Igκ antibody. (J) Co-IP validation of Igκ and MYLK interaction in transfected 293T cells. (K) In vitro pull-down assay confirming direct interaction between Igκ and MYLK. MYLK: myosin light chain kinase; WT: wild-type; cKO: conditional knockout; LC-MS/MS: liquid chromatography-tandem mass spectrometry.

To investigate the mechanism by which Igκ promotes liver regeneration, we conducted proteomics analysis with liver tissues from WT and cKO mice following 70% PHx. Mass spectrometry identified 79 upregulated and 210 downregulated proteins (**|** Fold Change**|** ≥ 2, *P*-value ≤ 0.05) (Figure 4A). GO enrichment analysis of these differentially expressed proteins revealed that Igκ deficiency disrupts cellular component organization, particularly affecting cytoskeleton-related process including cytoskeletal protein binding, actin binding, and actin cytoskeleton. Furthermore, KEGG pathway analysis highlighted enrichment of focal adhesion and ECM-receptor interaction, which were critical pathways for cytoskeletal remodeling among the deregulated proteins (Figure 4B). To validate these findings, we analyzed expression levels of key cytoskeletal components, including actin, myosin, microtubules, and intermediate filaments. Notably, all examined cytoskeletal elements except microtubule-associated proteins showed significant downregulation in cKO mice (Figure 4C). Given the essential role of cell proliferation in tissue regeneration, we also assessed the changes in proliferation-related proteins. Consistent with earlier *in vivo* observations, proteins associated with proliferation, including Cdk1, Mki67, and Pcna, as well as Mcm, were significantly reduced in Igκ-deficient mice (Figure 4D). These results indicate that Igκ promotes liver regeneration by modulating cytoskeletal integrity, suggesting a mechanism by which structural dynamics may drive proliferative recovery after injury.

### Igκ interacts with MYLK and inhibits its K48-linked ubiquitination to stabilize cytoskeletal dynamics

To explore whether Igκ directly regulates cytoskeletal organization during liver regeneration, we screened the interacting proteins of Igκ by immunoprecipitation-mass spectrometry (IP-MS) on liver tissue lysates from WT and cKO mice. The lysates were incubated with an anti-Igκ rabbit polyclonal antibody or rabbit IgG as an isotype control, followed by immunoprecipitation. The 15 potential interaction partners for Igκ were identified *via* LC-MS/MS analysis (Figure 4E). Venn diagram analysis identified seven overlapping proteins between Igκ interactors and downregulated proteins from our prior proteomic screen (Figure 4F & G). Functional annotation *via* the Uniprot database revealed MYLK, a kinase critical for cytoskeleton remodeling, as a top candidate. MYLK drives cytoskeletal reorganization through phosphorylating myosin light chain (MLC), thereby enhancing myosin-actin binding to promote cell contraction, motility, and migration.^[15,16]^ These processes are essential for tissue repair and regeneration. We confirmed the co-localization of Igκ with MYLK through confocal immunofluorescence in THLE-2 cells (Figure 4H) and validated endogenous interaction of Igκ with MYLK in mouse liver tissue lysates *via* co-IP (Figure 4I). To further identify the specific Igκ domain mediating MYLK binding, we generated truncated mutants targeting the variable (V) and constant (C) regions of Igκ. Co-IP assays demonstrated that the constant region of Igκ specifically binds to MYLK (Figure 4J). This interaction was further validated through *in vitro* pull-down assays using reciprocal truncation mutants of MYLK paired with Igκ domain variants (Figure 4K).

**Figure 5:**
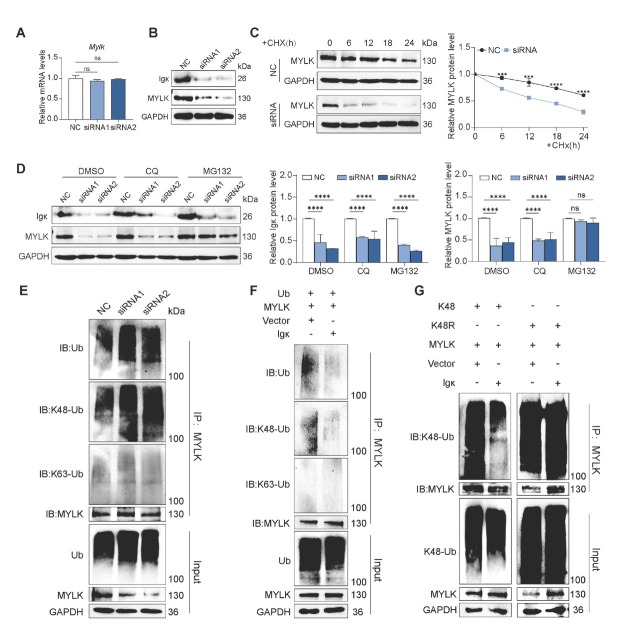
Igκ interacts with MYLK and suppresses its K48-linked polyubiquitination. (A) MYLK mRNA levels in Igκ-knockdown THLE-2 cells analyzed by RT-qPCR (*n* = 3). (B) MYLK protein levels in Igκ-knockdown THLE-2 cells assessed by western blot. (C) THLE-2 cells were transfected with Igκ siRNA or control siRNA, and then treated with CHX (50 μg/mL) at indicated time points. Western blot analysis was performed to evaluate MYLK protein levels and quantification of MYLK expression is summarized (right panel) (*n* = 3). (D) THLE-2 cells were transfected with Igκ siRNA or control siRNA, following with MG132 or CQ treatment. MYLK protein levels were assessed by western blot. Statistical results are shown on the right. (E) & (F) MYLK ubiquitination analysis in HEK-293T cells transfected with HA-tagged ubiquitin, MYLK and si-Igκ (E) or His-tagged Igκ (F) by western blot. (G) K48-specific ubiquitination of MYLK was evaluated by western blot in HEK-293T cells transfected with His-tagged K48-ubiquitin or K48R mutant ubiquitin, MYLK and Igκ as indicated, respectively. Data presented as the mean ± SD. ^***^P < 0.001, ^****^P < 0.0001, ns, not significant. MYLK: myosin light chain kinase; CHX: cycloheximide.

Subsequently, we explored the regulatory relationship between Igκ and MYLK. Quantitative PCR analysis revealed that targeted knockdown of Igκ expression in THLE-2 cells did not alter *MYLK* mRNA levels (Figure 5A). However, MYLK protein levels were significantly reduced upon siRNA-mediated Igκ knockdown in THLE-2 cells, suggesting post-translational regulation (Figure 5B). IHC analysis of DILI specimens showed concomitant upregulation of both Igκ and MYLK compared to controls (Figure 1A & S2), further supporting their functional coupling at the protein level. To determine the underlying mechanism, we assessed MYLK protein stability using cycloheximide (CHX) chase assays in THLE-2 cells. As shown in Figure 5C, Igκ knockdown significantly accelerated MYLK degradation. Treatment with the proteasome inhibitor MG 132 prevented this Igκ knockdown-induced reduction of MYLK protein levels in THLE-2 cells, whereas treatment with the lysosome inhibitor chloroquine (CQ) did not (Figure 5D). These findings collectively demonstrate that Igκ deficiency accelerates proteasome-dependent degradation of MYLK. Consistent with this mechanism, subsequent *in vitro* ubiquitination assays revealed that Igκ knockdown enhanced MYLK ubiquitination in THLE-2 cells (Figure 5E), while Igκ overexpression suppressed its ubiquitination (Figure 5F). Given the distinct functional roles of ubiquitin chains, where K48-linked modifications typically mediate proteasomal degradation, while K63-linked chains primarily modulate protein-protein interactions and kinase activation,^[17]^ we systematically interrogated the chain-type specificity of MYLK ubiquitination. Notably, Igκ knockdown enhanced K48-linked ubiquitination of MYLK, and its overexpression selectively attenuated this modification, with no concomitant changes observed in K63-linked ubiquitination patterns (Figure 5E & F). This regulatory effect was abolished through site-directed mutagenesis of K48-specific ubiquitination sites, confirming the requirement for canonical K48-specific ubiquitination machinery in MYLK regulation (Figure 5G). Collectively, these findings demonstrate that Igκ interacts with MYLK *via* its constant region, stabilizing the kinase by inhibiting K48-linked ubiquitination and subsequent proteasomal degradation, finally this interaction likely underpins the role of Igκ in maintaining cytoskeletal integrity during liver regeneration.

### Igκ maintains cytoskeletal stability *via* MYLK-dependent manner

**Figure 6:**
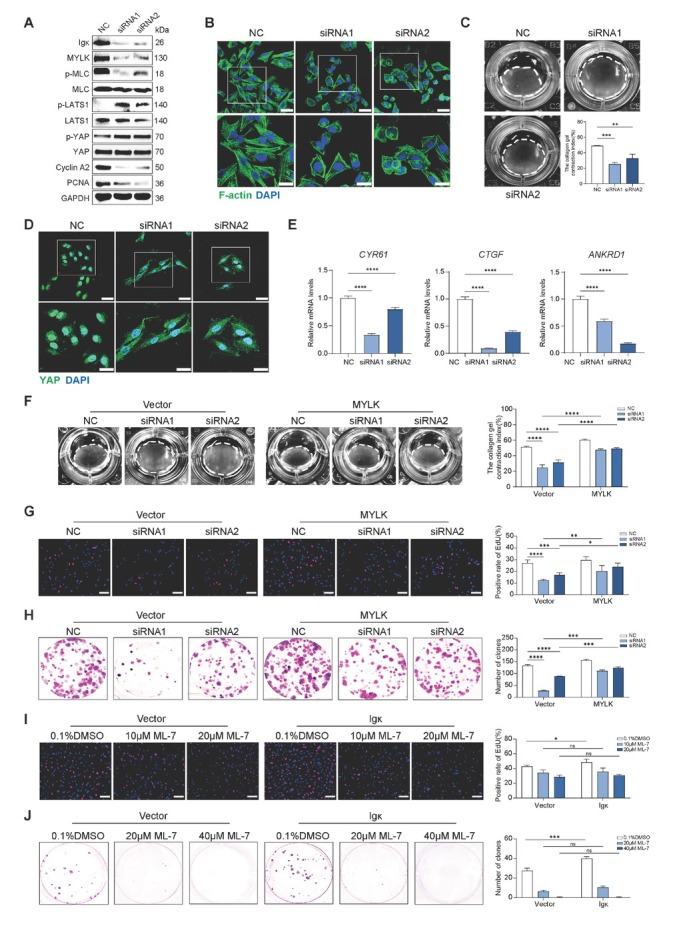
Igκ relies on MYLK to regulate cytoskeletal stability. (A) Western blot analysis detected MYLK, p-MLC, MLC, p-LATS1, LATS1, p-YAP, YAP, Cyclin A2 and PCNA levels after Igκ knockdown in THLE-2 cells. (B) Phalloidin staining after Igκ knockdown was performed to assess cytoskeletal structure in THLE-2 cells with fluorescence intensity analyzed by microscopy. (C) Cellular contractility was evaluated *via* CGCA. The contraction index (CI) was calculated as: CI = 1-(D/D_0_)^2^ × 100%, where D_0_ and D represent initial and final gel diameters. (D) Subcellular localization of YAP was analyzed by immunofluorescence after Igκ knockdown in THLE-2 cells. (E) The mRNA levels of YAP downstream target genes (*CYR61*, *ANKRD1* and *CTGF*) were analyzed by RT-qPCR in Igκ-knockdown THLE-2 cells (*n* = 3). (F) CGCA assessed contractile capacity of THLE-2 cells co-transfected with MYLK and si-Igκ. (G) & (H) Proliferation capacity was evaluated by EdU incorporation assay (G) and the colony formation assay (H) in THLE-2 cells co-transfected with MYLK and si-Igκ. Scale bar: 100 μm. Quantification of EdU-positive cell rate and colony numbers (*n* = 3) is shown on the right. (I) & (J) Proliferation capacity was assessed by EdU incorporation (I) and the colony formation assay (J) in THLE-2 cells treated with ML-7 (MYLK inhibitors) and Igκ. Scale bar: 100 μm. Quantification of EdU-positive cell rate and colony numbers (*n* = 3) is shown on the right. Data presented as the mean ± SD. ^*^P < 0.05, ^**^P < 0.01, ^***^P < 0.001, ^****^P < 0.0001. MYLK: myosin light chain kinase; YAP: Yes-associated protein; CGCA: collagen gel contraction assay; PCNA: proliferating cell nuclear antigen.

To investigate the functional interaction between Igκ and MYLK, we first knocked down Igκ in THLE-2 cells. Western blot analysis revealed a significant decrease in total MYLK, phosphorylated myosin light chain (p-MLC), and the cell proliferation markers Cyclin A2 and PCNA. Concurrently, there was a marked increase in phosphorylated LATS1 as a core kinase of the Hippo pathway and phosphorylated Yes-associated protein (p-YAP) (Figure 6A). Immunofluorescence staining further demonstrated diminished expression of cytoskeletal components, including F-actin (Figure 6B), CK18, and α-tubulin (Supplementary Figure S3A). Since MYLK-mediated MLC phosphorylation activates myosin II ATPase to drive actomyosin contractility,^[18,19]^ we assessed the contractile capacity of THLE-2 cells following Igκ knockdown using a collagen gel contraction assay (CGCA). The results revealed that Igκ knockdown significantly reduced collagen gel contraction, indicating impaired cellular contractility (Figure 6C). Furthermore, immunofluorescence staining showed impaired nuclear translocation of YAP, consistent with the observed increase in phosphorylated YAP levels (Figure 6D), as cytoplasmic retention of YAP typically correlates with its phosphorylation status. To determine the impact of reduced YAP activity following Igκ knockdown, we examined the expression of canonical YAP target genes by RT-qPCR analysis. As shown in Figure 6E, mRNA levels of *CYR61*, *CTGF*, and *ANKRD1* genes were significantly downregulated upon Igκ knockdown, consistent with a decrease in YAP-mediated transcription. To further define the role of MYLK in Igκ-mediated cytoskeletal and proliferative regulation, we conducted rescue experiments. Igκ knockdown disrupted cytoskeletal integrity, while exogenous MYLK restored contractility defects caused by Igκ knockdown through CGCA assays (Figure 6F). Similarly, the results of the EdU incorporation assay and the colony formation assay demonstrated that Igκ knockdown significantly inhibited THLE-2 cell proliferation, but this effect was reversed by MYLK overexpression (Figure 6G-H). These findings suggest that MYLK restoration mitigates cytoskeletal and proliferative deficits induced by Igκ loss. To further validate the necessity of MYLK in Igκ-driven cell proliferation, we inhibited MYLK activity in THLE-2 cells with ML-7, a specific inhibitor of MYLK. The results of the EdU incorporation assay and the colony formation assay demonstrated that ML-7 treatment significantly inhibited cell proliferation (Supplementary Figure S3B-D), and this inhibition persisted even under Igκ overexpression (Figure 6I-J), indicating that MYLK is indispensable for the pro-proliferative effect of Igκ. These results demonstrate that hepatocyte-derived Igκ sustains cytoskeletal stability by preserving MYLK protein levels, which in turn drives actomyosin contractility and cell proliferation, which is key processes for liver regeneration.

### Igκ supplementation rescues liver regeneration in cKO mice

**Figure 7:**
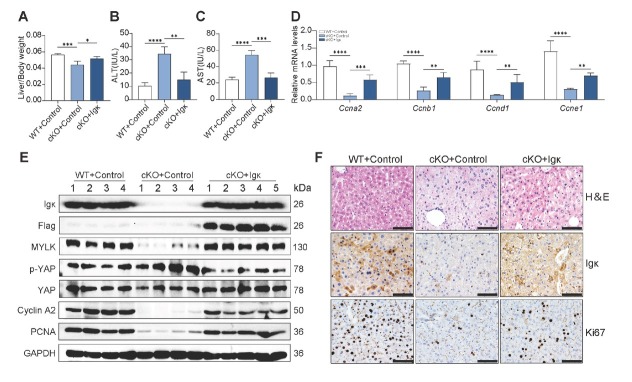
Igκ supplementation rescues liver regeneration in cKO mice. (A) - (C) Statistical analysis of Liver-to-body weight ratio (A) and serum ALT (B) and AST (C) levels. (D) Hepatic mRNA levels of cell cycle genes (*Ccna2*, *Ccnb1*, *Ccnd1* and *Ccne1*) analyzed by RT-qPCR. (E) Western blot analysis of Igκ, Flag-tagged Igκ, MYLK, p-YAP, total YAP, Cyclin A2 and PCNA in liver tissues of PHx mice following AAV-8-Igκ administration. (F) Representative images of H & E staining (upper) and IHC staining of Igκ and Ki67 (lower) of PHx mice following AAV-8-Igκ administration. Scale bars: 50 μm. Data presented as the mean ± SD. ^*^P < 0.05, ^**^P < 0.01, ^***^P < 0.001, ^****^P < 0.0001. MYLK: myosin light chain kinase; YAP: Yes-associated protein; PCNA: proliferating cell nuclear antigen; ALT: alanine aminotransferase; AST: aspartate aminotransferase; IHC: immunohistochemistry; cKO: conditional knockout.

To establish the therapeutic potential of Igκ in liver regeneration, we administered AAV8-Igκ vectors to cKO mice *via* tail vein injection. Two weeks post-injection, PHx was performed on WT and cKO mice, with liver tissues harvested at 72 h. Notably, the liver regeneration capacity of cKO mice was restored following AAV8-Igκ administration, and the liver-to-body weight ratio in AAV8-Igκ-treated cKO mice showed a marked increase compared to untreated cKO mice, although it did not fully recover to WT levels (Figure 7A). Concurrently, the severity of liver injury was alleviated, as evidenced by reduced serum ALT and AST levels (Figure 7B & C). In addition, the mRNA levels of cell-cycle-related genes (*Ccna2, Ccnb1, Ccnd1, and Ccne1*) were also restored (Figure 7D). The efficacy of AAV8-Igκ expression in mouse liver tissues was confirmed by western blotting and immunohistochemical staining, which demonstrated increased Flag and Igκ expression levels. Concurrently, the protein expression levels of MYLK, Cyclin A2, PCNA, and Ki67 were restored, while p-YAP levels were downregulated (Figure 7E & F). Furthermore, H & E staining revealed that liver injury was significantly alleviated in AAV8-Igκ-treated mice (Figure 7F). Collectively, these data demonstrate that exogenous Igκ functionally rescues regenerative defects in cKO mice, further establishing its necessity in hepatic proliferation and repair.

## Discussion

The liver is responsible for maintaining metabolism and detoxification in the body and is highly regenerative. Proper regeneration of hepatocytes is vital to prevent liver failure or mortality.^[1]^ In the present study, we first demonstrated that the Igκ expression was significantly elevated during the liver regeneration process and further confirmed that hepatocyte-derived Igκ markedly enhanced hepatocyte proliferation, thereby promoting liver regeneration *via* stabilizing cytoskeleton structure in a MYLK-dependent manner to trigger YAP signaling. Moreover, adenovirus-mediated delivery of Igκ alleviated liver injury repair and regeneration. Taken together, our findings reveal the role of hepatocyte-derived Igκ in liver regeneration and provide the structural basis for hepatocyte proliferation by maintaining the stability of MYLK proteins and ensuring the integrity of the cytoskeleton, indicating its potential as a drug for liver regeneration.

Prior research reveals that the expression of Igκ in hepatocytes was elevated during ConA-induced acute liver injury.^[14]^ While Igκ (including free Igκ light chains) is traditionally considered a plasma cell-derived surrogate marker for humoral inflammatory process,^[20]^ multiple groups have detected Igκ in B cell-deficient mice's cardiac, colon, lung, and kidney tissues, where they perform essential cellular biological function.^[12]^ Our study further demonstrated significant Igκ upregulation post-PHx, with its expression pattern aligning closely with that of the cell cycle protein Cyclin A2 and the proliferation markers PCNA and Ki67. Interestingly, a similar phenomenon was also detected in B cell-deficient mice. More importantly, liver biopsies from patients with DILI exhibited markedly higher Igκ protein levels compared to healthy controls. These findings collectively indicate that hepatocyte-derived Igκ likely plays a significant role in the regenerative response to acute liver injury.

To further elucidate the role of hepatocyte-derived Igκ, we established two distinct liver injury models using hepatocyte-specific Igκ knockout mice, including partial hepatectomy and acute APAP treatment.^[21]^ The liver exhibits extraordinary regenerative capacity post partial resection through robust hepatocyte proliferation to restore original mass and architecture while compensating for compromised hepatic function, establishing this model as a classical system for studying liver regeneration mechanisms over decades.^[22,23]^ In clinical practice, hepatic regeneration following DILI critically mediates functional recovery. For example, APAP intoxication triggers centrilobular hepatocellular necrosis and sterile inflammation (which may progress to ALF without intervention),^[24,25]^ followed by robust hepatocyte proliferation that replaces dead cells, thereby enabling spontaneous recovery.^[26]^ Our study found that, in both models, hepatocytic Igκ-deficient mice exhibited reduced liver-to-body weight ratios, elevated serum ALT/AST levels (which reflect the extent of liver damage), and impaired reparative hepatocyte proliferation compared to control mice. Furthermore, we found that Igκ overexpression promoted the proliferation and migration of the human hepatocyte cell line THLE-2 *in vitro*, whereas Igκ knockdown significantly suppressed these processes. These observations suggest that Igκ-promoted liver regeneration critically supports liver homeostasis and function.

Recent mechanistic studies have found that hepatocyte-derived Igκ mediates cellular resistance to ConA-induced liver injury by the mitochondrial death pathway, which might be related to the inhibition of the NF-κB signaling pathway and activation of JNK *via* the cytoskeleton dynamic.^[14]^ Moreover, intracellular Vκ4–1/Jκ3-Igκ might interact with plectin as an intermediate filament-related protein, participating in the assembly of intermediate filaments and microfilaments, thereby functioning as a cytoskeleton-associated protein to support cell survival.^[12]^ Consistent with prior findings, our study revealed that Igκ directly interacted with MYLK, which plays a key role in inducing ATPase-driven actin-myosin contraction by phosphorylating the regulatory light chain of myosin II.^[27]^ Phosphorylated myosin II is an important effector of cytoskeletal activities in many cellular functions.^[28]^ In non-muscle cells, Ser 19 phosphorylation of myosin II correlates with stress fiber formation, capping of cell surface receptors, and cytokinesis.^[29, 30, 31, 32]^ It has been reported that the deletion of MYLK leads to a reduction in membrane strength and F-actin filaments, and MYLK deficiency not only weakens the synthesis of various transmembrane complexes such as myosin II, integrins, and fibronectin but also disrupts membrane tension and protrusions, resulting in instability of the cytoskeleton.^[15,33,34]^ Of note, the deletion of MYLK significantly suppresses the proliferation and migration of liver cancer cells.^[35]^ Our findings demonstrate that ML-7, a MYLK-specific inhibitor, significantly suppressed the proliferation and migration of THLE-2 cells. Notably, this suppression persisted even upon Igκ overexpression, whereas reintroduction of MYLK rescued the proliferation deficits induced by Igκ knockdown. In addition, markedly increased expression of MYLK was observed in liver tissues of individuals diagnosed with DILI. Based on these results, we conclude that Igκ might modulate hepatocyte proliferation *via* MYLK signaling.

Liver regeneration primarily relies on hepatocyte proliferation, initiated by hepatic microenvironmental cues such as mechanical and chemical sensors.^[1]^ In particular, mechanosensors can reorganize the actomyosin network to regulate nuclear morphology and transcription to drive proliferation. In the regenerating liver, actomyosin network organization critically regulates hepatocyte migration and proliferation, whereas its instability disrupts mechanotransduction, thereby compromising injury response and regenerative capacity.^[5]^ In our study, Igκ knockdown significantly downregulated multiple cytoskeleton-related proteins, including F-actin, α-tubulin, and CK18. More importantly, the absence of Igκ induces MYLK degradation, thereby reducing its stability and downregulating the levels of p-MLC, ultimately impairing the actomyosin network. Notably, the administration of AAV-8-Igκ effectively restored the liver regeneration ability *via* the MYLK axis in cKO mice, providing compelling evidence that Igκ is indispensable for liver regeneration following injury.

YAP pathway is a key signaling pathway that regulates proliferation and mechanotransduction *via* cytoskeletal dynamics.^[36]^ Phosphorylated YAP/TAZ undergo cytoplasmic sequestration and degradation, whereas unphosphorylated YAP/TAZ translocates into the nucleus and activates the Transcriptional Enhancer Associate Domain (TEAD) transcription factors to upregulate genes involved in liver repair and regeneration.^[37,38]^ It has been demonstrated that F-actin dynamics plays a pivotal role in maintaining hepatic homeostasis in mammals by regulating the YAP mechanotransduction pathway.^[39]^ F-actin accumulation has been shown to suppress LATS1 phosphorylation, promote YAP nuclear translocation, and initiate downstream gene expression.^[40]^ During the process of liver regeneration, YAP nuclear levels transiently rise to initiate proliferation and decline upon completion to regulate hepatocyte proliferation and reorganization.^[5]^ Recent investigations have revealed that mechanical stretching enhances hepatocyte proliferation during liver regeneration through YAP translocation and subsequent upregulation of heparin-binding epidermal growth factor (HB-EGF).^[6]^ In our study, we observed that Igκ deficiency reduced F-actin levels, which promotes LATS1 phosphorylation and thereby activates the Hippo pathway. Consequently, YAP nuclear translocation and downstream gene expression are suppressed. This demonstrates that Igκ maintains liver regeneration *via* regulating MYLK within actomyosin-driven YAP signaling axis. This mechanism establishes the structural foundation essential for hepatocyte proliferation and actively supports the liver's regenerative capacity.

From a translational clinical perspective, our findings position Igκ as a potential therapeutic target for enhancing liver regeneration in chronic liver injury. A major clinical challenge in managing conditions such as advanced fibrosis or cirrhosis is the inherent impairment of hepatic regenerative capacity. Current strategies primarily focus on removing the underlying etiological insult, with limited options available to directly promote parenchymal repair and regeneration. The association we observed between Igκ expression and regenerative responses following liver injury provides a rationale for exploring Igκ and its associated pathways as targets for pro-regenerative therapies. The potential could be explored through several innovative therapeutic modalities. For instance, our findings may inform the development of liver-targeted mRNA-based therapeutics. By leveraging the identified role of Igκ, future studies could design mRNA constructs that modulate this pathway or its key effectors, encapsulated within delivery systems optimized for hepatocyte uptake. Such strategies aim to directly enhance hepatocyte survival and proliferation, offering a complementary approach to etiology-focused treatments. To realize this translational promise, subsequent research must delineate the precise mechanistic role and upstream pathway of Igκ within hepatocytes. Collectively, our work not only underscores the biological relevance of Igκ in liver regeneration but also provides a conceptual framework for developing novel regeneration-oriented treatment strategies for chronic liver injury.

## Conclusions

Overall, our findings underscore the critical role of hepatocyte-derived Igκ in liver regeneration. Mechanistically, Igκ stabilizes MYLK by suppressing ubiquitination-mediated degradation, thereby preserving cytoskeletal integrity. These findings establish a link between cytoskeletal dynamics and hepatocyte proliferation, advancing our understanding of liver regeneration mechanisms and highlighting Igκ's potential as a promising therapeutic candidate in the field of liver regeneration.

## Supplementary Material

Supplementary Material Details
