# Supplementary material for: Immunoglobulin κ drives liver regeneration by stabilizing MYLK to maintain cytoskeletal integrity and promote hepatocyte proliferation
Source: J Transl Int Med. 2026 Jun 13;14(3):398–412. doi: 10.1515/jtim-2026-0054 (PMC13320523; doi:10.1515/jtim-2026-0054)
Supplement: Supplementary file 1 — Supplementary Material Details [file jtim-2026-0054_sm.pdf]

Supplementary materials

Materials and methods

Cell culture

The THLE-2 cell line (RRID: CVCL_3803), along with its proprietary culture medium, was purchased in 2023 from Pricella Biotechnology Co., Ltd. (Wuhan, China). The HEK293T cell line (RRID: CVCL_0063), originally sourced from the American Type Culture Collection (ATCC), was provided by the Peking University Center of Human Disease Genomics. HEK293T cells were cultured in Dulbecco's Modified Eagle Medium (DMEM; Invitrogen, USA) supplemented with 10% fetal bovine serum (FBS; PAN Biotech, Germany) and 1% penicillin-streptomycin (HyClone, USA). All cells were maintained at 37°C in a humidified condition with 5% CO_2. None of the cell lines listed above were contaminated.

Cell transfection

Small interfering RNAs (siRNAs) were synthesized by GenePharma (Shanghai, China). The His/myc-tagged Igκ was cloned into the pcDNA3.1 vector, while the Flag-tagged pCMV-MYLK plasmid was obtained from Magen Biotechnology (Guangzhou, China). Transfection was performed using Lipofectamine 3000 (Invitrogen, USA) for siRNA delivery and polyethyleneimine (PEI; Polysciences, USA) for plasmid DNA transfection, following the manufacturer's protocols. The siRNA sequences are as follows: Igκ-siRNA1(5'-AGGCCAAAGUACAGUGGAA-3') and Igκ-siRNA2(5'-GACAGCACCUACAGCCUCATT-3').

Cell proliferation assay

Cell proliferation and viability were assessed using the Cell Counting Kit-8 (CCK-8;

Dojindo Molecular Technologies, Japan) following the manufacturer's protocol. For colony formation assays, transfected cells were seeded in 12-well plates at a density of 400 cells per well and cultured for 7-10 days. Colonies were stained with 1% crystal violet solution for 20 minutes at room temperature (RT) and manually counted. The EdU incorporation assay was performed using a commercial kit (Beyotime Biotechnology, China), and proliferation rates were calculated based on the percentage of EdU-positive cells.

Cell migration assay

Cell migration was evaluated using 8μm pore size polycarbonate membrane inserts (Corning, USA) in a 24-well Transwell system. Briefly, 1.5×10^5 transfected cells in 250μL serum-free medium were seeded in the upper chamber, while 750μL complete medium supplemented with 10% FBS was added to the lower chamber as a chemoattractant. After 48 hours of incubation at 37°C with 5% CO_2, cells on the membrane were fixed and stained with 1% crystal violet for 20 minutes at RT. Migrated cells were quantified by counting under an inverted microscope using Image J software.

Serum biochemistry

Blood samples were collected from the mouse models, and serum levels of ALT and AST were measured using standard enzymatic assays according to the manufacturer's instructions (C009-2-1 and C010-2-1; Nanjing Jiancheng Bioengineering Institute, Nanjing, China).

RNA extraction and quantitative real-time PCR assay

Total RNA was extracted from tissues and cells using TRIzol™ Reagent (Invitrogen, USA).

cDNA was synthesized from 2 µg of total RNA using the Revert Aid First Strand cDNA Synthesis System (Thermo Fisher Scientific, Waltham, MA, USA) according to the manufacturer's protocol. Quantitative real-time PCR was performed with Hieff® qPCR SYBR® Green Master Mix (Yeasen Biotechnology, Shanghai, China), and gene expression was quantified using the $2^{-\Delta\Delta Ct}$ method, with GAPDH as the endogenous normalization control. Primer sequences, synthesized by Sangon Biotech Co., Ltd. (Shanghai, China), are provided in Supplementary Table S1.

Immunohistochemistry (IHC)

Liver tissues were fixed in 10% neutral-buffered formalin and embedded in paraffin. Tissue sections were deparaffinized in xylene, dehydrated through a graded ethanol series, and subjected to antigen retrieval in 10 mM citrate buffer (pH 6.0) using a heating device. Endogenous peroxidase activity was quenched by incubating the sections in 3% hydrogen peroxide for 10 minutes, followed by blocking with goat serum at RT for 30 minutes. Sections were incubated with primary antibodies at 4°C overnight and then with HRP-conjugated anti-mouse/rabbit IgG at RT for 30 minutes. Immunoreactivity was visualized using an enhanced diaminobenzidine (DAB) kit (Dako, Denmark), and nuclei were counterstained with hematoxylin.

Western blot analysis

Protein lysates were prepared from liver tissues or cultured cells using ice-cold RIPA lysis buffer (Beyotime Biotechnology, Shanghai, China) containing 1% protease inhibitor cocktail and phosphatase inhibitors (Roche, Basel, Switzerland). Protein extracts were quantified using a BCA protein assay (Thermo Fisher Scientific, Waltham, MA, USA). Equal

amounts of protein were loaded in SDS-PAGE and transferred to nitrocellulose Membranes (Amersham Biosciences, Little Chalfont, UK). Membranes were blocked with 5% non-fat milk or 1 hour at RT, followed by incubation with primary antibodies overnight at 4°C. After washing with TBST, membranes were incubated with appropriate HRP-conjugated secondary antibodies for 1 hour at RT. Protein bands were visualized using an enhanced chemiluminescence (ECL) kit (Thermo Fisher Scientific, Waltham, MA, USA) and quantified using an iBright™ CL750 imaging system (Thermo Fisher Scientific, Waltham, MA, USA). All antibodies used are listed in Supplementary Table S2.

Immunofluorescence (IF) staining

Cells were seeded on poly-L-lysine-coated glass coverslips (NEST Biotechnology Co., Ltd., Wuxi, China) in 12-well culture plates at a density of 5×10^4 cells/well. Upon reaching 70%-80% confluency, cells were fixed with freshly prepared 4% paraformaldehyde (PFA) in PBS for 20 minutes at RT, followed by permeabilization with 0.3% Triton X-100 (Solarbio Science & Technology Co., Ltd., Beijing, China) for 15 minutes. After blocking with 10% goat serum for 30 minutes, cells were incubated with primary antibodies diluted in blocking buffer overnight at 4℃ in a humidified chamber. Following three PBS washes, samples were incubated with fluorophore-conjugated secondary antibodies for 1 hour at RT in the dark. Phalloidin (PF00001) was obtained from Wuhan Sanying Biotechnology Co., Ltd. (Wuhan, China). Antibody sources and catalog numbers are documented in Table S2. Nuclei were counterstained with DAPI in antifade mounting medium (Vector Laboratories, Burlingame, CA, USA). Confocal images were acquired using an Olympus FV3000 laser scanning microscope.

Co-immunoprecipitation (Co-IP)

For Co-IP assays, liver tissue lysates were prepared in lysis buffer (50 mM Tris, 150 mM NaCl, 1% NP-40, pH 7.5) and incubated with anti-Igκ antibody or isotype control overnight at 4°C with gentle rotation. Immunoprecipitation was performed using protein G beads at 4°C for 4 hours. After extensive washing to remove nonspecifically bound proteins, the immunoprecipitates were analyzed by western blotting with the indicated antibodies.

For Co-IP assays in HEK293T cells, protein lysates were prepared using the same lysis buffer. The lysates were incubated with anti-His-tag monoclonal antibody-conjugated magnetic beads (MBL International, Woburn, MA, USA) at 4°C for 2 hours. Following washing with wash buffer (50 mM Tris-HCl, 150 mM NaCl, 0.1% NP-40), the precipitates were subjected to western blot analysis.

Pull-down assay

His-tagged truncated mutants of Igκ variable and constant regions were expressed using the TNT®T7 Quick Coupled Transcription/Translation System (Promega Corporation, Madison, WI, USA). Non-specific binding sites in the in vitro translation products were blocked with 4% bovine serum albumin (BSA) at 4°C for 30 minutes. The translation products were then incubated with GST-tagged truncated mutants of MYLK (Sino Biological Inc., Beijing, China) and GST beads (GE Healthcare, Chicago, IL, USA) at 4 for 2 hours. Samples were washed twice with buffer I (PBS containing 0.5% NP-40) and once with buffer II (PBS containing 0.5% NP-40 and 200 mM KCl). After extensive washing, the protein complexes were eluted by boiling in 2×loading buffer and analyzed by western

blotting.

Label-free proteomics analysis

Total proteins were extracted from liver tissues and analyzed by liquid chromatography-tandem mass spectrometry (LC-MS/MS) using an Orbitrap Exploris 480 mass spectrometer, following standard protocols at the Institute of Biotechnology, Peking University Health Science Center (Beijing, China). Proteins exhibiting significant differential expression ($|\text{Fold Change}| \geqslant 2$, p-value $\leqslant 0.05$) were identified using DESeq2. Subsequently, these differentially expressed proteins were subjected to functional annotation through KEGG pathway and Gene Ontology (GO) enrichment analyses. Visualization of the enrichment results was performed using R software.

Figures and Tables

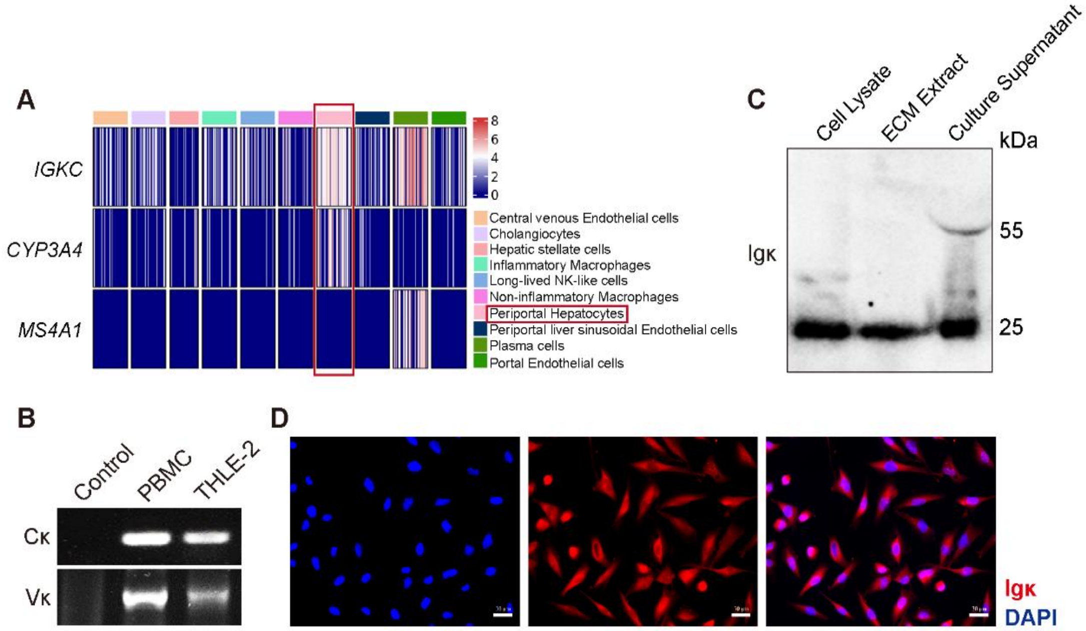

Supplementary Figure S1. Igκ-FLC is expressed in hepatocytes. (A) Analysis of single-cell RNA sequencing data (GSE115469) from scLiverDB database (http://bioinfo.life.hust.edu.cn/liverdb) revealed detectable expression of IGKC in hepatocytes. CYP3A4 and MS4A1 were used to label hepatocytes and plasma cells, respectively. (B) RT-PCR confirmed the presence of IGK transcripts in the immortalized human hepatocyte cell line THLE-2, with peripheral blood mononuclear cell (PBMC) serving as a positive control. (C) Western blot analysis demonstrated Igκ-FLC expression in THLE-2 cells. (D) Immunofluorescence staining of Igκ within THLE-2 cells. Scale bars, 50μm.

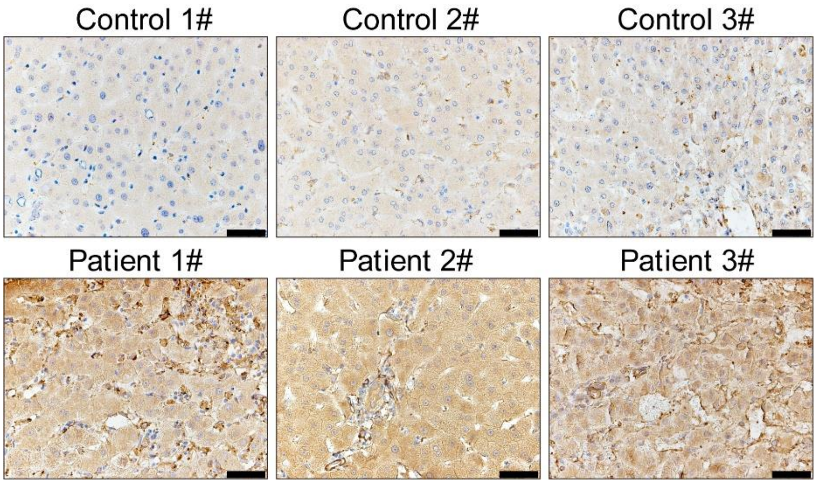

Supplementary Figure S2. Representative immunohistochemical staining of MYLK expression in DILI and non-tumor normal liver tissues. Scale bars: 50μm.

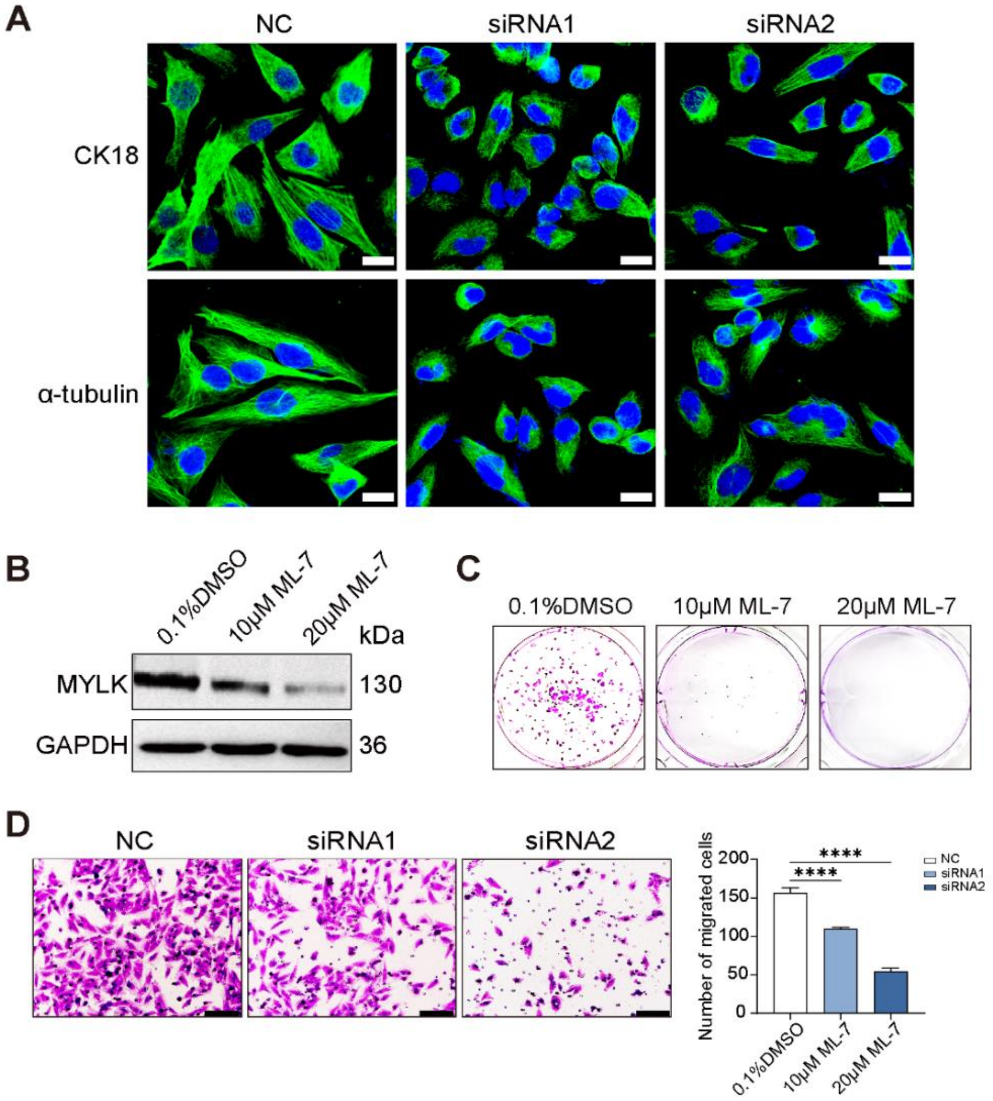

Supplementary Figure S3. Treatment with ML-7 inhibits proliferation and migration of THLE-2 cells. (A) Immunofluorescence analysis of CK18 and α-tubulin expression after Igκ knockdown in THLE-2 cells. Scale bar: 20μm. (B) Western blot analysis of MYLK protein levels in ML-7-treated THLE-2 cells. (C) Colony formation assay assessing the proliferation capacity of THLE-2 cells after ML-7 treatment. (D) Transwell migration assay evaluating the migration ability of THLE-2 cells after ML-7 treatment. Quantification of migrated cells is shown (right) (n = 3). Scale bar: 100μm. Data presented as the mean ± SD. **** $p < 0.0001$.

Supplementary Table S1. The sequence of primers used for qPCR

Gene name		Primer sequence 5’-3’
Ccna2	Forward primer	ACAGAGTGTGAAGATGCCCTGGCT
	Reverse primer	AGCATGTGGTGATTCAAAACTGCCA
Ccnb1	Forward primer	CAATTATCGGAAGTGTCGGATCA
	Reverse primer	CTGGTGAACGACTGAACTCCC
Ccnd1	Forward primer	GCGTACCCTGACACCAATCTC
	Reverse primer	ACTTGAAGTAAGATACGGAGGGC
Ccne1	Forward primer	GTGGCTCCGACCTTTCAGTC
	Reverse primer	CACAGTCTTGTCAATCTTGGCA
MYLK	Forward primer	GCTGAACGGAAAGACCCTCA
	Reverse primer	CTTCTCGATGGAGACGGAGC
GAPDH	Forward primer	CAAGGTCATCCATGACAACTTTG
	Reverse primer	GTCCACCACCCTGTTGCTGTAG

Supplementary Table S2. The list of antibodies

Antigen	Product code	Supplier	RRID
α-Tubulin	80762-1-RR	Proteintech	AB_2918911
CK18	10830-1-AP	Proteintech	AB_2133164
Cyclin A2	18202-1-AP	Proteintech	AB_10597084
Flag-Tag	66008-2-Ig	Proteintech	AB_2881492
GAPDH	TA-08	ZSGB-Bio	AB_2747414
GST- Tag	M20007	Abmart	AB_2864360
His-Tag	66005-1-Ig	Proteintech	AB_11232599
Igκ	14678-1-AP	Proteintech	AB_1851235
Ki67	27309-1-AP	Proteintech	AB_2756525
K48-Ubiquitin	8081	Cell Signaling Technology	AB_10859893
K63-Ubiquitin	5621	Cell Signaling Technology	AB_10827985
MLC	sc-365243	Santa Cruz Biotechnology	AB_10848269
MYLK	ab76092	Abcam	AB_1524000
PCNA	10205-2-AP	Proteintech	AB_2160330
p-MLC	AP0955	ABclonal	AB_2863864
p-YAP	57706	Cell Signaling Technology	AB_2799531
Ubiquitin	3936	Cell Signaling Technology	AB_331292
YAP	sc-101199	Santa Cruz Biotechnology	AB_1131430
p-LATS1	9157	Cell Signaling Technology	AB_2133515
LATS1	9153	Cell Signaling Technology	AB_2296754
